# Contribution of the West African Health Organization to the prevention and control of the Mpox outbreak in West Africa two weeks after the declaration as a public health emergency of international concern

**DOI:** 10.11604/pamj.2024.49.21.45296

**Published:** 2024-09-23

**Authors:** Sombie Issiaka, Lokossou Kuassi Virgil, Sani Ali, Usman Aishat Bukola, Keita Namoudou, Diallo Ely, Agbla Felix, Aissi Athanase Melchior

**Affiliations:** 1 West African Health Organization, BP153, Bobo-Dioulasso, Burkina Faso

**Keywords:** Mpox outbreak, preparedness and response, regional coordination, health emergency, West African Health Organization

## To the Editors of the Pan African Medical Journal

In West Africa, the Economic Community of West African States (ECOWAS) established the West African Health Organization (WAHO) in 1987 to address health challenges in the region [1]. In response to the 2014-2016 Ebola Virus Disease outbreak, the ECOWAS Regional Centre for Disease Surveillance and Control (RCSDC) was created in 2016 under WAHO's supervision to enhance coordination in preparedness and response to epidemics and other health emergencies [2]. During the COVID-19 pandemic, WAHO and RCSDC played pivotal roles in regional coordination, highlighting their importance in addressing health crises [3-6]. On August 14, the World Health Organization (WHO) declared Mpox a “public health emergency of international concern [7].” A day earlier, the Africa Centers for Disease Control and Prevention declared a continent-wide public health emergency, reflecting the gravity of the situation [8]. By August 25, Mpox had affected 12 African countries, with 5,281 cases and thirty-two deaths. As of 3^rd^ September, the West African region contributed ninety-eight cases and one death across four countries: Nigeria, Liberia, Côte d´Ivoire, and Guinea. While West Africa was not as severely affected as Central and East Africa, urgent public health measures were needed.

On August 15, following the declarations, WAHO organized an emergency meeting under the leadership of its Director General. The meeting evaluated the epidemiological situation and devised a regional action plan to prevent, detect, and respond to the Mpox outbreak. Two crucial decisions emerged: first, WAHO would issue a situation report on the Mpox epidemic, including prevention strategies, and share it with national health officials in the region. Second, a high-level coordination meeting involving ECOWAS Health Ministers would be convened to discuss the outbreak and formulate a regional preparedness and response plan. This meeting activated WAHO´s incident management system, which aligned with pre-established Standard Operating Procedures approved by ECOWAS member states (MS).

On August 16, RCSDC produced a regional situation report on Mpox, which was disseminated to all stakeholders. WAHO distributed a readiness assessment tool to MS to evaluate their preparedness for Mpox. Preliminary findings showed that MS had about 50% of the necessary capacities to manage Mpox effectively. These early assessment reports played a crucial role in shaping the regional response.

On August 22, WAHO organized an online meeting with representatives from 13 ECOWAS MS, which included seven Health Ministers, two secretaries-general, and other key health officials. The meeting served as a platform for MS to share information on their responses to Mpox, such as reinforcing surveillance, case investigations, and enhancing laboratory capacities. Ministers also identified their needs, which included diagnostic reagents, collaborative surveillance efforts, capacity building, and improvements in infection prevention and control which is in line with WHO recommendations [9,10]. During the meeting, WAHO´s Director General announced a funding allocation of USD 1.6 million to support initial response activities in the region. From August 22 to 24, 2024, ECOWAS “One Health” national platform focal points conducted a tabletop simulation exercise in Dakar, Senegal, aimed at improving preparedness for Mpox. The exercise allowed participants to review and evaluate multi-sectoral collaboration mechanisms, drawing on lessons learned from previous outbreaks. Discussions focused on surveillance, joint investigations, managing contact cases, and exchange of best practices.

To further strengthen laboratory capacity, WAHO collaborated with the Institute Pasteur de Dakar and WHO to organize a training session for thirty-five laboratory personnel from MS between August 26 and 30 on Mpox diagnostics and sequencing, equipping participants with the necessary skills to detect the virus. After the training, each country received diagnostic and sequencing kits, ensuring they had the resources to detect Mpox cases promptly. WAHO engaged regional donors and partners such as the United States Agency for International Development, the West Africa Regional Office, and the World Bank's Health Security Program to support Mpox response efforts.

On August 27, a regional meeting was held with National Public Health Institutes in MS where Nigeria, Côte d'Ivoire, and Liberia shared their experiences on the Mpox outbreak, offering insights into effective response strategies. WAHO presented a draft regional response plan and diagnostic approach during this meeting, with technical guidance from the Institute Pasteur. It was agreed that participants would reconvene in two weeks to share further updates and experiences.

WAHO´s efforts extended beyond regional boundaries, as it participated in a preparatory meeting at the continental level during the 74^th^ session of the WHO Africa Regional Committee. This meeting brought together Health Ministers across the continent to discuss priorities for the Mpox response, including vaccination. WHO reiterated its commitment to collaborating with regional institutions to bolster laboratory capacities through training.

## Conclusion

The rapid activation of political leadership, technical expertise, and financial support by WAHO demonstrates its ability to coordinate regional efforts. This experience underscores the importance of regional economic communities in delivering coordinated responses to health crises, positioning them as vital players in future efforts to prevent and control public health emergencies.
